# Laser Fragmentation‐Induced Defect‐Rich Cobalt Oxide Nanoparticles for Electrochemical Oxygen Evolution Reaction

**DOI:** 10.1002/cssc.201903186

**Published:** 2019-12-30

**Authors:** Mingquan Yu, Friedrich Waag, Candace K. Chan, Claudia Weidenthaler, Stephan Barcikowski, Harun Tüysüz

**Affiliations:** ^1^ Department of Heterogeneous Catalysis Max-Planck-Institut für Kohlenforschung Kaiser-Wilhelm-Platz 1 45470 Mülheim an der Ruhr Germany; ^2^ Center for Nanointegration Duisburg-Essen (CENIDE) University of Duisburg-Essen Duisburg 47057 Germany; ^3^ Institute of Technical Chemistry I University of Duisburg-Essen Essen 45141 Germany; ^4^ Materials Science and Engineering School for Engineering of Matter, Transport and Energy Arizona State University Tempe Arizona 85287 USA

**Keywords:** electrocatalysis, metal oxides, nanostructures, oxygen evolution reaction, structural defects

## Abstract

Sub‐5 nm cobalt oxide nanoparticles are produced in a flowing water system by pulsed laser fragmentation in liquid (PLFL). Particle fragmentation from 8 nm to 4 nm occurs and is attributed to the oxidation process in water where oxidative species are present and the local temperature is rapidly elevated under laser irradiation. Significantly higher surface area, crystal phase transformation, and formation of structural defects (Co^2+^ defects and oxygen vacancies) through the PLFL process are evidenced by detailed structural characterizations by nitrogen physisorption, electron microscopy, synchrotron X‐ray diffraction, and X‐ray photoelectron spectroscopy. When employed as electrocatalysts for the oxygen evolution reaction under alkaline conditions, the fragmented cobalt oxides exhibit superior catalytic activity over pristine and nanocast cobalt oxides, delivering a current density of 10 mA cm^−2^ at 369 mV and a Tafel slope of 46 mV dec^−1^, which is attributed to a larger exposed active surface area, the formation of defects, and an increased charge transfer rate. The study provides an effective approach to engineering cobalt oxide nanostructures in a flowing water system, which shows great potential for sustainable production of active cobalt catalysts.

## Graphical abstract

**Smash it up**: Sub‐5 nm cobalt oxide nanoparticles are produced by pulsed laser fragmentation in liquid (PLFL) in a flowing water system. Significantly larger surface area, crystal oxidation, and the formation of structural defects (Co^2+^ defects and oxygen vacancies) are evidenced through the PLFL process, leading to a superior catalytic activity for the oxygen evolution reaction.

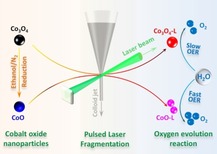

## Introduction

As the energy crisis and environmental pollution increase, public interest and research topics have increasingly focused on developing a sustainable energy conversion/storage system with high efficiency and economical scalability.^1^ Water splitting provides a promising path for converting renewable energy into hydrogen, a clean fuel that can be consumed for various energy demands.^2^ The anodic half reaction, the oxygen evolution reaction (OER), is very challenging and has been considered as the bottleneck for overall water splitting as a large overpotential is needed to drive this thermodynamically uphill reaction of four‐proton‐coupled electron transfer.^3^ To lower the energy barrier, catalyzing OER requires active electrocatalysts for competitive energy conversion efficiency. However, most active OER electrocatalysts are still based on noble metal/metal oxides (e.g., Pt, RuO_2_, and IrO_2_), which limits their large‐scale application for water splitting considering the prohibitive price and elemental scarcity of noble metals.3a, 4 Therefore, it is crucial to design efficient catalysts based on abundant transition metals that may be feasible for commercial mass manufacture.

Owing to their low cost and 3d electron configuration, first‐row transition metal based catalysts have been intensively studied as alternatives to noble metal catalysts,^5^ in various forms such as oxides,5d, 6 perovskites,3b (oxy)hydroxides,5c, 7 phosphates,^8^ and metal–organic frameworks.^9^ Of these materials, spinel Co_3_O_4_ is particularly interesting, owing to its: (1) high catalytic activity,3a (2) satisfactory stability under reaction conditions,^10^ and (3) applicability over a broad range of pH conditions.^11^ When employed as the electrocatalyst for OER, which occurs on the surface, the catalytic activity of Co_3_O_4_ can be improved by nanostructuring, which is a straightforward strategy to provide more active sites with higher surface area. Different synthetic approaches have thus been developed to prepare nanostructured Co_3_O_4_, such as hydrothermal methods,^12^ nanocasting,5e and plasma engraving.^13^ Moreover, theoretical calculations have revealed that tuning the electronic state of Co_3_O_4_ modulates the adsorption energy of intermediate species to lower the energy barrier.^14^ This can be achieved by substitution of a foreign element (e.g., Ni, Cr, Cu),5c, 15 defect formation,^13^, ^16^ and constructing hybrid structures.^17^ Despite the massive amount of work on developing cobalt oxide catalysts, there is still a challenge to fabricate cobalt oxide in a scalable system, which delivers nanoscale morphology with modified electrical structure for efficient OER catalysis.

Recently, laser‐induced engineering of materials has been adopted to effectively reduce particle sizes with a narrow size distribution.^18^ As no surfactants are required during the process, with simply pure water employed as the solvent, the products from laser synthesis expose a clean surface without the surface‐blocking effect of molecular ligands or residues of chemical precursors.^18^ In addition, high temperature and pressure can be generated locally upon laser irradiation, followed by a rapid cooling down of the products. Such a process normally induces formation of defects and structural disorder in the obtained product, resulting in not only reduced particle size but also modulated electronic states.^18^, ^19^ A large range of starting materials could be subjected to laser irradiation for efficient structural engineering, including metal colloids, ionic crystal powders, and semiconductors.^18^ Remarkably, this technology is appealing for material synthesis owing to its simple preparation process compared with chemical synthesis.^18^, ^20^ Therefore, laser irradiation is a desirable technology to prepare cobalt oxide based electrocatalysts for OER. In a pioneering work, nanoparticles with broad size distributions from nanometers to micrometers were produced by pulsed laser fragmentation in liquid (PLFL) of cobalt and cobalt oxides (CoO and Co_3_O_4_) powders dispersed in different solvents.^20^ Whereas Co_3_O_4_ was obtained for all educt powders during PLFL in water, the crystal structure remained unchanged during PLFL in hexane. Subsequent studies focused on a water‐based PLFL approach to produce Co_3_O_4_ particles with a controlled particle size for applications in electrochemical catalysis.19a, 21 However, these reported PLFL approaches took place in a stationary container, for example, a beaker, with up to hours of irradiation using nanosecond‐pulsed laser beams, which hindered its applicability in terms of practical material production. Recently, some of us reported a synthesis method to treat commercial CoFe_2_O_4_ powder by employing PLFL.^22^ Reduced particle size and structural disorder were induced on CoFe_2_O_4_ particles that were dispersed in water and irradiated in a flowing system by a picosecond‐pulsed laser beam. As discussed, when water is used as a solvent, the laser‐irradiated product can be obtained with clean surfaces without any organic residues. By tuning the energy intensity of the laser, the particle concentration, and the flow rate of the colloid, the physicochemical properties (band gap,^23^ crystal phase,^22^ and atomic disorder^23^) of the targeted material can be tuned. In addition, ultrashort (picosecond)‐pulsed laser beams allow a more efficient size reduction compared with longer, nanosecond‐pulsed laser beams owing to reduced heat loss.^24^ As this PLFL technique is processed in a flowing system, continuous production of fragmented catalysts for large‐scale synthesis is feasible. Further upscaling of the process can be done by using a laser system with higher average powers. Consequently, this continuous‐flow PLFL approach shows distinct advantages over batch PLFL owning to these factors: (1) energy dose control on particles, (2) shorter irradiation period, and (3) continuous production, making it applicable for sustainable production processes of nanomaterials.^18^, ^22^, ^25^

In this work, we utilized PLFL in a water‐flowing system to engineer the structure of cobalt oxides (CoO and Co_3_O_4_) for electrochemical OER. Spinel Co_3_O_4_ was initially prepared by a facile method with the utilization of coffee waste (CW) as a sustainable hard template.^15^ Subsequently, a mild reduction procedure was applied to obtain CoO while maintaining the mesoporous structure comprised of approximately 8 nm particles. The subsequent PLFL treatment induced particle downsizing along with crystal oxidation in water on CoO, although no significant changes were observed when the starting oxide had a spinel phase. Particle fragmentation (from ca. 8 nm to ca. 4.2 nm) of CoO is proposed to be induced by the oxidation process in water, where instant temperature elevation upon laser irradiation‐initiated fragmentation. An increased specific surface area was thus exhibited for the fragmented CoO particles, as well as the formation of Co^2+^ defects and oxygen vacancies owing to a fast heating/quenching process. It was then found out that the obtained cobalt oxide with sub‐5 nm nanoparticles could provide more catalytic sites and enable faster electron transfer when serving as an OER electrocatalyst. As a result, the fragmented and oxidized cobalt oxide shows a much higher OER activity compared with those of the initial oxides and also the ordered mesoporous Co_3_O_4_ that were nanocast from ordered mesoporous silica.

## Results and Discussion

**Scheme 1:**
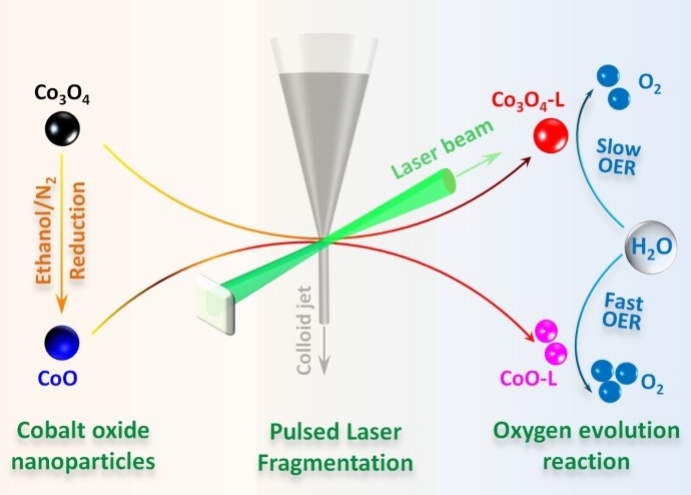
Preparation of cobalt oxide electrocatalysts by pulsed laser fragmentation in water.

The synthetic strategies to obtain the cobalt oxide particles are illustrated in Scheme 1. First, spinel Co_3_O_4_ was prepared from a scalable CW‐templating method.^15^ Through a simple impregnation–calcination process, Co_3_O_4_ nanoparticles with diameters of approximately 8 nm were obtained (see the Supporting Information, Figure S1). Along with the decomposition of metal precursors to crystalize Co_3_O_4_, the CW template was removed during the same calcination step in the presence of oxygen (see the Supporting Information for the experimental details). Distinct reflections in the X‐ray diffraction (XRD) patterns of the CW‐templated oxides are well indexed to the spinel structure of Co_3_O_4_, with Co^2+^ and Co^3+^ located at tetrahedral and octahedral sites, respectively (Figure S2). Then, the Co_3_O_4_ was exposed to a mild reduction step under ethanol vapor at 270 °C to obtain cobalt monoxide (CoO). The XRD patterns of the reduced oxide exhibited three reflections at 36.5°, 42.4°, and 61.5°, which can be assigned to the (1 1 1), (2 0 0), and (2 2 0) planes of cubic CoO, respectively. Afterwards, the CW‐templated Co_3_O_4_ and CoO were irradiated with a picosecond laser beam in suspension. The products were collected after drying and labeled as Co_3_O_4_‐L and CoO‐L, respectively. When Co_3_O_4_, which is a thermodynamically stable phase, was irradiated as the target material, the XRD patterns for the product (Co_3_O_4_‐L) were similar to those for the starting material. In contrast, when CoO was irradiated with the laser beam, the XRD results show an oxidation process from cubic CoO to spinel Co_3_O_4_ with broader peak widths, demonstrating that the PLFL process led to an oxidation and crystal downsizing effect on CoO. It should be noted that non‐degassed, deionized water was utilized as the solvent for the PLFL process. Dissolved molecular oxygen may have contributed to the oxidation of CoO. In addition, laser‐induced splitting of water molecules during PLFL is known to lead to the formation of reactive oxygen species.^20^, ^26^

**Figure 1:**
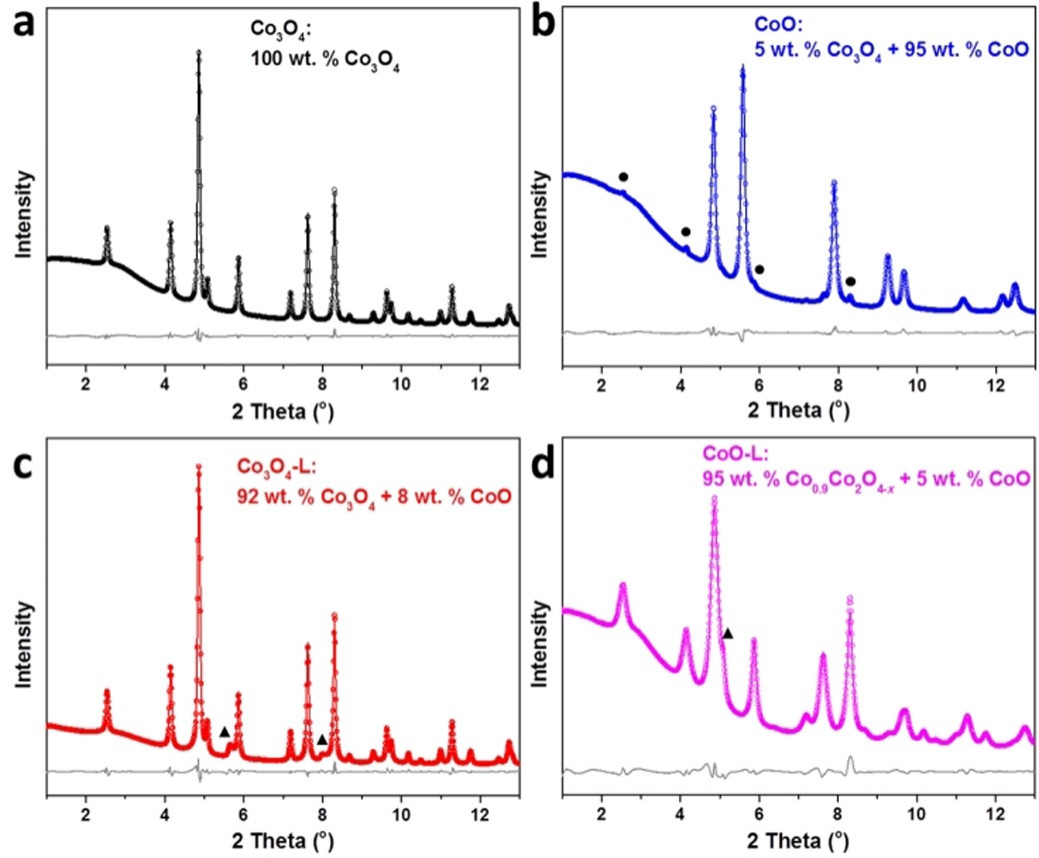
Rietveld refinement analysis of synchrotron diffraction patterns of CW‐templated Co_3_O_4_ (a), Co_3_O_4_‐L (b), CoO (c), and CoO‐L (d). The reflections marked by the circles and triangles are indexed to spinel Co_3_O_4_ and cubic CoO, respectively.

To confirm the oxidation process and obtain more accurate information on the crystal structure, high‐resolution XRD patterns were measured by using synchrotron radiation. As starting models for structure refinements, the crystal structures given in a previous study were used.^27^ The results from the Rietveld analysis are listed in Table S1. The as‐prepared CW‐Co_3_O_4_ sample was phase pure with the standard spinel structure, whereas a mixture of Co_3_O_4_ (5 wt %) and CoO (95 wt %) phases was observed in the ethanol‐reduced sample, confirming the reduction effect from the ethanol treatment (Figure 1 a,b). It should be noted that laboratory XRD using CuK_α1/2_ radiation is not able to detect such a low concentration of Co_3_O_4_ (5 wt % or 1.61 mol %). Cobalt‐containing samples are excited by the Cu radiation, which causes fluorescence radiation. This also increases the noise of the measured data and is one reason that cobalt phases of low content cannot be seen clearly. After the PLFL process, similar XRD patterns were observed on Co_3_O_4_‐L compared with pristine Co_3_O_4_, apart from a small amount of CoO generated owing to laser radiation (Figure 1 c). The formation of CoO was also observed when treating commercial CoFe_2_O_4_ with the same PLFL process, with thermal decomposition proposed as the reason.^22^ For CoO‐L, substantial oxidation had occurred in the sample, with only 5 wt % of the CoO phase remaining (Figure 1 d). More importantly, the oxidized phase was determined by structure refinement to be Co^2+^_0.9_Co^3+^_2_O^2−^_4−*x*_, which possesses 10 % fewer Co^2+^ cations in the tetrahedral sites of the perfect spinel structure, suggesting the formation of Co^2+^ defects as well as oxygen vacancies. It has been reported that instantaneous temperature elevation upon picosecond laser irradiation can initiate the formation of vacancy defects, including metal and oxygen vacancies, which become trapped in the crystal with subsequent quenching in water.^18^, 19b, 28

Owing to the lower formation energy of Co^2+^ defects than that of Co^3+^ defects, Co^2+^ defects are preferentially generated in a Co_3_O_4_ spinel structure,^16^ matching well with the observation in this study. It has been well documented that introducing vacancies is favorable for both electrocatalytic activity and structural stability. The formation of defects results in distortion to neighboring atoms and electronic delocalization. Such effects can enhance the charge transport in semiconductors and lower the adsorption energy of water molecules, respectively, which contribute to a higher electrocatalytic activity.^16^, ^18^, ^19^, ^29^ Furthermore, the structure distortion helps to decrease the surface energy and hence endow the defective Co_3_O_4_ with better structural stability.^16^, ^30^

**Figure 2:**
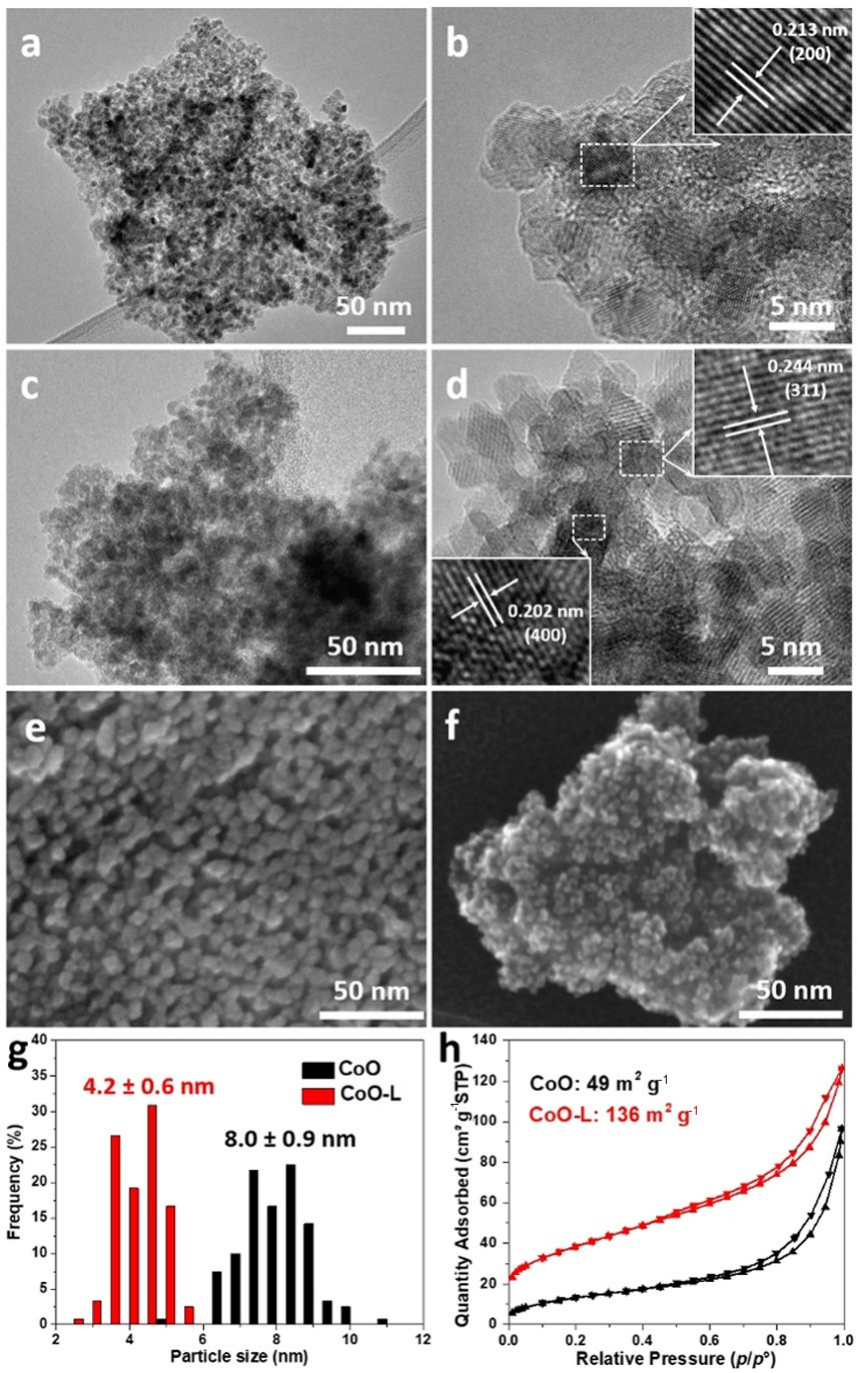
TEM (a, c), high‐resolution TEM (b, d), and SEM (e, f) of CoO and CoO‐L, respectively. (g) Particle size distribution histograms. (h) Nitrogen sorption isotherms. Insets in (b) and (e) are the corresponding close‐up of the marked rectangles (white color) showing the lattice fringes.

The particle size and morphology were then examined by transmission and scanning electron microscopy (TEM, SEM). Consistent with the XRD result, a similar morphology was exhibited for Co_3_O_4_‐L, where nanoparticles of approximately 8 nm were interconnected to form a mesoporous structure (Figure S3). Owing to the mild reduction conditions (270 °C for 2 h), particles were not grown or aggregated on CoO, as shown in Figure 2 a. A closer examination at higher magnification confirms the high crystallinity with clear lattice fringes (Figure 2 b). The space between lattice fringes was measured to be 0.213 nm and 0.246 nm, corresponding to the (2 0 0) and (1 1 1) planes of cubic CoO, respectively. After the PLFL process, much smaller particles were formed on CoO‐L, as shown in Figure 2 c, compared with the TEM image of the initial CoO. In Figure 2 d, the observed lattice fringes in the CoO‐L crystallites correspond to planes in spinel Co_3_O_4_, supporting the XRD result on the laser‐induced oxidation effect. Meanwhile, vacancy sites (marked with yellow circles in Figure S4) were observed in the crystal structure, suggesting the laser‐induced formation of defects on CoO‐L. This is in line with the Rietveld analysis of XRD patterns obtained by synchrotron measurements. To check the fragmentation effect of PLFL, the average particle size was then calculated based on 120 particle counts (Figure S5). As seen in Figure 2 g, the average particle size of 8 nm was dramatically decreased to 4.2 nm for CoO after laser irradiation. Furthermore, SEM images provide a visualized downsizing effect comparing the particles of CoO (Figure 2 e) and CoO‐L (Figure 2 f), which were interconnected to form a mesoporous structure.

Normally, particle size reduction contributes to a higher specific surface area, which can be examined by nitrogen physisorption measurements. The mesoporous structure of these oxides was confirmed from the typical type IV isotherms (Figure 2 h and Figure S6). The same Brunauer–Emmett–Teller (BET) surface area of 46 m^2^ g^−1^ was measured for Co_3_O_4_ and Co_3_O_4_‐L, slightly lower than that of CoO (49 m^2^ g^−1^). CoO‐L showed a very high BET surface area of 136 m^2^ g^−1^, agreeing well with the XRD and TEM results. For OER catalysts, such a high surface area is highly desirable as more catalytic sites on the surface could be exposed to the electrolyte.

**Figure 3:**
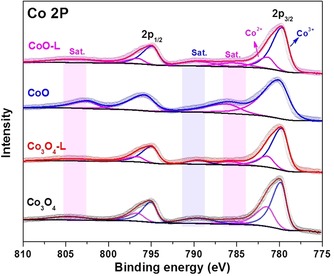
Co 2p XPS spectra of Co_3_O_4_, Co_3_O_4_‐L, CoO, and CoO‐L.

To investigate the surface chemical state, X‐ray photoelectron spectroscopy (XPS) was carried out to study the oxidation state of atoms in the top few layers. The binding energies of fitted peaks in the Co 2p and O 1s regions are summarized in Tables S2 and S3, respectively. In the high‐resolution Co 2p XPS spectra (Figure 3), characteristic peaks of Co^2+^ and Co^3+^ were fitted fo the Co 2p_3/2_ and Co 2p_1/2_ peaks of Co_3_O_4_, Co_3_O_4_‐L, and CoO‐L,^31^ in good agreement with the XRD result that these oxides mainly show the spinel structure of Co_3_O_4_. The peaks at approximately 779.7 and 794.9 eV are ascribed to Co^3+^, with the peaks located at about 781.4 and 796.6 eV corresponding to Co^2+^.19a, 29, 31, 32 For the reduced oxide CoO, the main peaks at 780.1 and 795.8 eV were assigned to only Co^2+^, accompanied by satellite peaks at 786.1 and 802.5 eV.^33^

In the O 1s spectra, four characteristic peaks of oxygen components were identified (Figure S7). Two intense peaks at 530.0 and 531.0 eV are assigned to the lattice O from the metal oxide and hydroxide, respectively.^31^ The other two minor peaks at 531.8 and 532.8 eV are due to the surface contamination of hydroxyl and carbonate ions from water and carbon dioxide.^31^, ^34^ The area of the lattice hydroxide peaks increased in the laser‐irradiated oxides, indicating cobalt hydroxide species were formed on the surface during the PLFL process. This could be due to the hydration of the hot oxide particles in water.^35^ Furthermore, Fourier transform infrared spectroscopy (FTIR) spectra were collected to study the generated species on the particles. Two sharp absorption bands at 547 and 655 cm^−1^, assigned to the Co‐O vibration of Co_3_O_4_, were observed on the spinel oxides (Figure S8).^36^ Compared with the pristine oxides, CoO‐L had much stronger peaks at 3340 and 1640 cm^−1^, which are attributed to the surface hydroxyl groups.19a, 36 The surface cobalt hydroxide species could be oxidized to oxyhydroxide species under anodic potential, which were regarded as active phase for OER.^37^ Therefore, the formation of cobalt hydroxide is beneficial for the electrocatalytic performance of oxides upon laser irradiation, in addition to its superior conductivity over that of crystalline Co_3_O_4_.^38^

The above‐mentioned characterization reveals the various effects of the PLFL process on Co_3_O_4_ and CoO. The laser irradiation leads to a particle downsizing, change of oxidation state, and formation of structural defects on cobalt monoxide (CoO), whereas the laser‐irradiated cobalt spinel oxide was generally unchanged. Although the mechanism of size reduction is still unclear during PLFL, it is generally believed that photothermal evaporation or Coulomb explosion induce the fragmentation.^18^, ^39^ Whereas lattice temperatures above the boiling point of the material need to be reached to initiate its photothermal evaporation, Coulomb explosion is the result of an extremely fast heating of the electrons of the material above a critical temperature, even higher than the boiling point of the material.^18^, ^40^ To achieve fragmentation by photothermal evaporation or Coulomb explosion, enough energy should be accumulated on the sample upon laser irradiation. The accumulated energy is mainly determined by the laser fluence, and the optical and thermal properties of target particles. In the case of the fragmentation of cobalt oxide particles, a previous study calculated the temperature rise in commercial Co_3_O_4_ as a function of laser fluence and found that a beam fluence of at least 410 mJ cm^−2^ was required to heat the commercial particles to boiling (above 3700 °C) upon 20 min of continuous irradiation in a stationary tube.19a Although a higher laser fluence (700 mJ cm^−2^) was applied in our system, a much lower temperature increase should be expected owing to: (i) a much smaller absorption cross section of the 8 nm particles compared with the commercial particles with an average diameter of around 400 nm, (ii) a significantly shorter laser pulse duration of 10 picoseconds compared with 7 nanoseconds, and (iii) the more rapid cooling of the particles in the flowing water system. As a result, Co_3_O_4_ particles were probably not fragmented because the local temperature could not reach the boiling point, and the spinel structure was maintained as it is the thermodynamically preferred crystalline phase.^22^ Nevertheless, the formation of a small amount of CoO suggests that the laser irradiation elevated the local temperature to the decomposition temperature of the Co_3_O_4_ phase (ca. 900 °C).^41^ In the case of cobalt monoxide (CoO), we propose that the fragmentation effect is related to the crystal transformation. The crystal structure of CoO is not stable at high temperature and it can be oxidized to Co_3_O_4_ spinel above 200 °C.^42^ During the laser treatment, cobalt monoxide was oxidized to cobalt spinel oxide by dissolved molecular oxygen or by reactive oxygen species, which can be products of laser‐induced water splitting.^20^ The oxidation step leads to the formation of polycrystalline Co_3_O_4_ and probably also to the size reduction. It remained unclear from the results of the analysis which mechanism the size reduction followed. However, a passivation of the surface of the size‐reduced Co_3_O_4_ particles by hydroxide species most likely prevented them from ripening.

To examine the mechanism of the size reduction more in detail, the cobalt oxides were irradiated with two orders of magnitude lower laser fluence (7 mJ cm^−2^) and their structural changes were characterized. The oxidation effect was found to similarly occur on cobalt monoxide, whereas Co_3_O_4_ remained as the spinel structure when using the lower energy laser irradiation (Figure S9). However, owing to relatively milder reaction conditions, a porous structure with smaller surface area was exhibited for CoO after the PLFL process using lower laser fluence (Figure S10). In addition, we conducted PLFL (700 mJ cm^−2^) on large cobalt oxide particles prepared through direct calcination of cobalt nitrate precursor. Severe changes including oxidation and particle fragmentation were only observed on particles with a crystal phase of CoO, further supporting the hypothesis that particle fragmentation goes along the oxidation route. Furthermore, we conducted ultraviolet‐visible spectroscopy on both large and nanosized cobalt oxide powders. The obtained results also supported that the oxidation effect is the key factor triggering effective particle fragmentation regardless of particle size. A detailed discussion on the comparison experiment can be found in the Supporting Information (Figures S11–S16).

**Figure 4:**
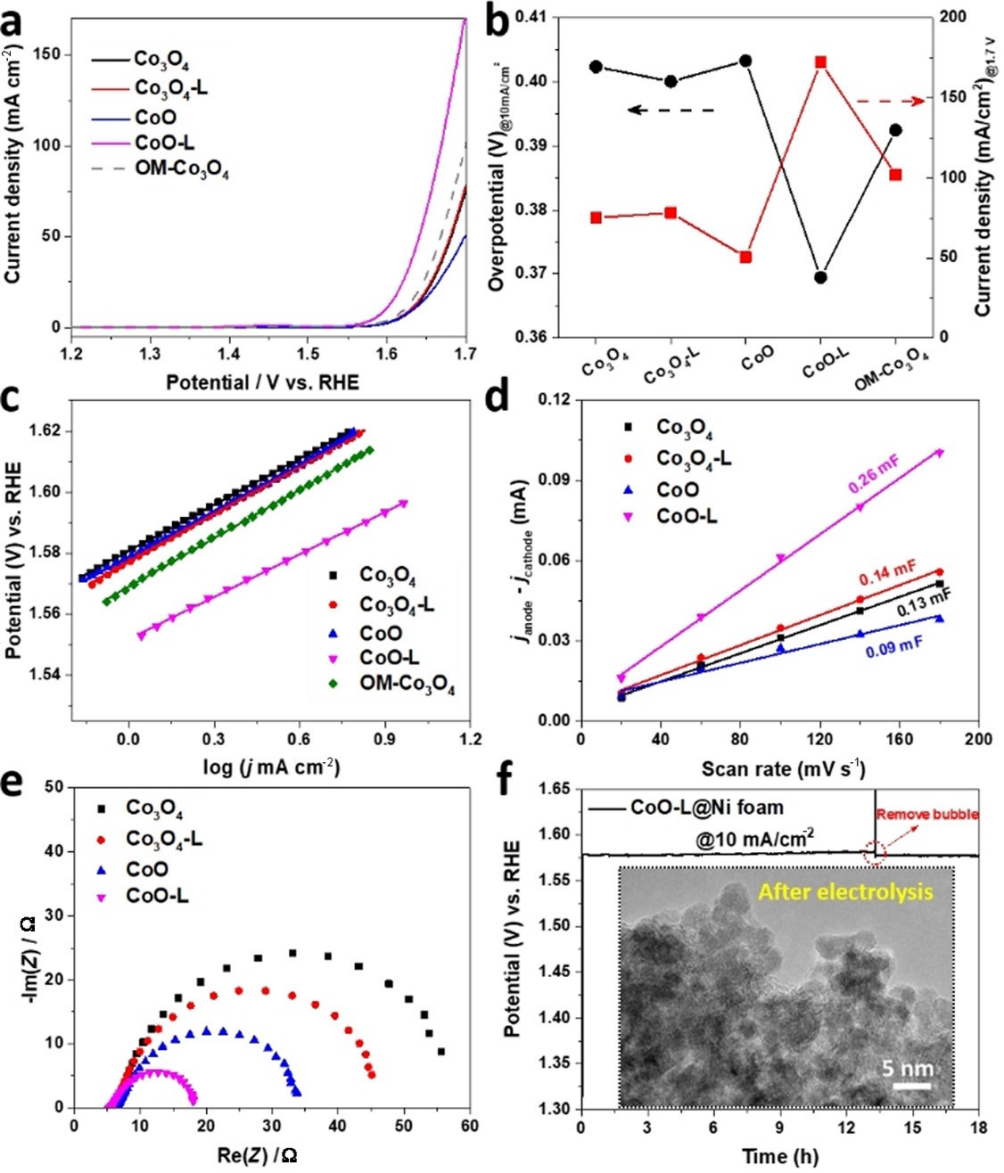
(a) The LSV curves of various cobalt oxides. The current density was determined by the geometry surface area of the glassy carbon electrode (0.196 cm^2^). (b) Comparison of the overpotential required to reach 10 mA cm^−2^ (left axis) and the current density at 1.7 V vs. RHE (right axis). (c) Tafel plots of cobalt oxides derived from their LSV curves correspondingly. (d) Capacitive current differences (Δ*j*=*j*_anode_−*j*_cathode_) at 1.05 vs. RHE against different scan rates. (e) The Nyquist plots measured at 1.6 V vs. RHE. (f) Chronopotentiometric curve of CoO‐L at a current density of 10 mA cm^−2^ and the inset shows a TEM image of CoO‐L scratched from the electrode after long‐term electrolysis.

The OER catalytic performance of the oxides was measured in 1 m KOH by following the protocol proposed by Jaramillo and co‐workers,3a with the electrochemical results listed in Table S4. As a reference catalyst, ordered mesoporous Co_3_O_4_ was prepared through nanocasting (labeled as OM‐Co_3_O_4_), and its structural characterization is presented in Figure S17. As shown in the linear sweep voltammetry (LSV) curves (Figure 4 a), OM‐Co_3_O_4_ exhibited higher OER activity over CW‐templated Co_3_O_4_, on account of its larger surface area (113 m^2^ g^−1^ vs. 46 m^2^ g^−1^). As predicted, the highest OER activity was observed for CoO‐L, which even outperformed the model catalyst (OM‐Co_3_O_4_). For a straightforward comparison, Figure 4 b summarizes the overpotential and the current density of these cobalt oxides electrocatalysts. To reach a current density of 10 mA cm^−2^, an overpotential of 369 mV is required for CoO‐L, which is considerably smaller than those of Co_3_O_4_ (402 mV), Co_3_O_4_‐L (400 mV), CoO (403 mV), and OM‐Co_3_O_4_ (392 mV). Additionally, significantly higher current density of CoO‐L was achieved at an applied voltage of 1.7 V (vs. reversible hydrogen electrode, RHE), implying that oxygen was generated much faster compared with that from other cobalt oxide electrocatalysts under the same potential.

The catalytic kinetics of the cobalt oxides were evaluated by Tafel plots, which were derived directly from the LSV curves, as shown in Figure 4 c. The calculated Tafel slope of CoO‐L is 46 mV dec^−1^, which is lower than those of other cobalt oxides (ca. 52 mV dec^−1^). Despite the same active species (cobalt hydroxide/oxyhydroxide) formed on all these cobalt oxides, the high porosity of CoO‐L allows for superior mass transport, which contributes to its lower Tafel slope (Table S4).^15^, ^43^ In addition, the formation of structural defects should also be taken into consideration, as defect‐induced electronic delocalization could optimize the adsorption energy for the OER intermediates.^16^, 19a A lower Tafel slope suggests more favorable OER kinetics,^44^ which is especially desirable for practical applications as the current increases dramatically with applying a higher voltage to the electrode. Based on the values of the two most important parameters of OER activity, that is, overpotential at 10 mA cm^−2^ and the Tafel slope, we find that CoO‐L displays a competitive catalytic performance compared with the benchmark cobalt electrocatalysts (Table S5).

Next, we employed different electrochemical approaches to illustrate the outstanding OER activity of CoO‐L. The catalytic performance is determined by the active site density of a catalyst. The double‐layer capacitance (*C*_dl_) was measured by using cyclic voltammetry (CV) to estimate the electrochemical surface area (ECSA) of the cobalt oxide catalysts.^45^ The CV curves were collected in a non‐Faradic region with different scan rates (Figure S18). As shown in Figure 4 d, the capacitance current differences were plotted against the scan rate, where the slope of the plot is proportional to the value of *C*_dl_. The largest *C*_dl_ was obtained on CoO‐L (0.26 mF), which was significantly higher than those for Co_3_O_4_ (0.13 mF), Co_3_O_4_‐L (0.14 mF), and CoO (0.09 mF). By dividing the specific capacitance of the metal oxide (0.04 mF cm^−2^),^45^ the corresponding ECSA were obtained (Table S4). The largest ECSA on CoO‐L resulted from the highly porous structure and provided more active sites exposed to the electrolyte for catalyzing electrochemical OER. Next, the LSV curves were normalized based on ECSA to compare the intrinsic activity of cobalt oxides. The largest normalized current density was obtained with CoO‐L (Figure S19), suggesting the amount of exposed active sites is not the sole promotor for OER activity.

As another key parameter to evaluate an electrocatalyst, the charge transfer rate of the cobalt oxides was measured by performing electrochemical impedance spectroscopy (EIS) at an overpotential of 350 mV (Figure 4 e). The corresponding Nyquist plots can be fitted into a simplified Randles circuit^45^, ^46^ (Figure S20). A similar resistance (ca. 6 Ω) under high frequency was observed for all the cobalt oxides, which is assigned to the electrolyte resistance (1 m KOH solution). The diameter of the semi‐circle is related to the charge transfer resistance (*R*_ct_), with the lower values indicative of faster charge transport kinetics. In comparison with the initial oxides, the samples after laser irradiation display lower *R*_ct_, which may be attributed to the formation of structural defects.19a Specifically, the *R*_ct_ of Co_3_O_4_ (48.8 Ω) was slightly decreased to 38.3 Ω, whereas a dramatic drop was exhibited in the value of *R*_ct_ from CoO (25.8 Ω) to CoO‐L (12 Ω). The different changes in *R*_ct_ can be explained by the contribution of the much smaller particles to a higher conductivity in CoO‐L. The reduction treatment led to a lower resistance on Co_3_O_4_ as well, in line with the other studies that reported that cobalt monoxide has a faster charge transfer rate than that of cobalt spinel oxide.^47^ Among these cobalt oxides, the lowest *R*_ct_ of CoO‐L enables much faster transfer of electrons from catalytic sites to the glassy carbon electrode, improving the reaction rate of OER.

Beside an efficient catalytic performance, the operating stability of an OER catalyst is essential to its application, especially in terms of practical water electrolysis. A stability test was carried out by using chronopotentiometry on CoO‐L loaded onto conducting Ni foam (1 mg cm^−2^, Figure 4 f). During delivery of a static current density of 10 mA cm^−2^ for 13 h, a slight increase in potential was observed, which is due to partial blocking of the catalyst surface by evolved oxygen. After removing the oxygen bubble, the potential dropped immediately and stayed constant for the following electrolysis. In addition, post‐testing characterization was done to check the structural stability of catalyst. As shown in the TEM image of the catalyst after electrolysis (inset of Figure 4 f), ultra‐small particles were maintained in the CoO‐L catalyst without severe particle growth or aggregation. These results suggest the robust durability of CoO‐L as an OER catalyst.

## Conclusions

A facile and sustainable production process was designed to engineer the structure of cobalt oxides through the PLFL process. Particle fragmentation was initiated on the reduced oxides and proceeded alongside crystal oxidation in water where the oxidative species were present and the local temperature was elevated under laser irradiation. Interestingly, relatively mild PLFL conditions seemed to be sufficient to initiate the size reduction. Fragmented oxides with a high surface area of 136 m^2^ g^−1^ were obtained, along with the formation of structural defects such as Co^2+^ and oxygen vacancies. Employed as OER electrocatalysts, the laser‐irradiated CoO‐L composed of sub‐5 nm particles provides more catalytically active sites and enables faster charge transfer, which were confirmed by electrochemical surface area analysis and impedance spectroscopy, respectively. As a result, significantly higher OER activity was exhibited with laser‐irradiated cobalt oxide, outperforming pristine and ordered mesoporous Co_3_O_4_ from nanocasting. Furthermore, satisfactory catalytic durability and structural stability were demonstrated on conducting substrates during long‐term water electrolysis. This work demonstrates the possibility of constructing ultrasmall nanoparticles in a water‐flowing system through the continuous, scalable PLFL process, which should be attractive for the economical production of active metal oxide catalysts.

## Experimental Section

### Synthesis of coffee waste‐templated Co_3_O_4_ and bulk Co_3_O_4_

The coffee waste‐templated Co_3_O_4_ was prepared through a simple impregnation–calcination procedure, and bulk Co_3_O_4_ was obtained directly from calcination of Co(NO_3_)_2_ **⋅**6 H_2_O. The calcination for both oxides was conducted at 400 °C for 4 h with a ramping rate of 2 °C min^−1^ under air. Detailed synthesis procedures can be found in our recent report.^15^

### Synthesis of CoO

CoO was prepared by reducing Co_3_O_4_ under ethanol atmosphere. In detail, nitrogen gas (100 mL min^−1^) was employed as carrier gas and bubbled through a 250 mL round‐bottomed flask filled with ethanol (120 mL). The gas mixture of N_2_ and ethanol was then purged through a tube, where a quartz boat filled with Co_3_O_4_ was located inside. The tube furnace was heated to 270 °C and kept at this temperature for 2 h before it was naturally cooled down under a N_2_ atmosphere. The ramping rate was 5 °C min^−1^ for the heating.

### Pulsed laser fragmentation in liquid (PLFL)

PLFL was conducted by using the second harmonic (532 nm) of two different picosecond‐pulsed (10 ps) Nd: YAG laser systems: a high‐power laser (PX 400‐3‐GH, EdgeWave, Würselen, Germany) and a low‐power laser (Atlantic, Ekspla, Vilnius, Lithuania). The pulse repetition rate was 100 kHz. Whereas the PX 400‐3‐GH provided a pulse energy of 500 μJ, the Atlantic laser was used at 5 μJ. Detailed information on the setup was described in our previous report.^22^ Aqueous suspensions of the different cobalt oxide powders had concentrations of 0.01 vol %. Liquid jets of the suspensions crossed the laser beam in focal position, in which energy densities of 700 mJ cm^−2^ (PX 400‐3‐GH) and 7 mJ cm^−2^ (Atlantic) were reached. The irradiated volume of colloids was achieved at 86.5 vol % after each passage. Thus, in principle, a high yield of 99.8 % could be obtained after three passages on the sample before drying. Owing to experimental limitations, for instance, reflection effects at the interface of the liquid jet and the surrounding air, the average energy densities were reduced by 27 % (PX 400‐3‐GH) and 38 % (Atlantic), as determined by differential measurements of the laser power. The details of electrochemical measurements and characterization are provided in the Supporting Information.

## Conflict of interest

*The authors declare no conflict of interest*.

## Supporting information

As a service to our authors and readers, this journal provides supporting information supplied by the authors. Such materials are peer reviewed and may be re‐organized for online delivery, but are not copy‐edited or typeset. Technical support issues arising from supporting information (other than missing files) should be addressed to the authors.

Supplementary
