# Supplementary material for: Laser Fragmentation‐Induced Defect‐Rich Cobalt Oxide Nanoparticles for Electrochemical Oxygen Evolution Reaction
Source: ChemSusChem. 2019 Dec 30;13(3):520–8. doi: 10.1002/cssc.201903186 (PMC7028056; doi:10.1002/cssc.201903186)
Supplement: Supplementary file 1 — Supplementary [file CSSC-13-520-s001.pdf]

CHEMISTRY & SUSTAINABILITY
CHEMSUSCHEM
ENERGY & MATERIALS

Supporting Information

Laser Fragmentation-Induced Defect-Rich Cobalt Oxide Nanoparticles for Electrochemical Oxygen Evolution Reaction

Mingquan Yu,[a] Friedrich Waag,[b, c] Candace K. Chan,[a, d] Claudia Weidenthaler,[a] Stephan Barcikowski,[b, c] and Harun Tüysüz*[a]

cssc_201903186_sm_miscellaneous_information.pdf

Electrochemical Measurements

Electrochemical measurements were performed in a three-electrode configuration using a rotating disc electrode (Model: AFMSRCE, PINE Research Instrumentation); a hydrogen reference electrode (HydroFlex, Gaskatel) and Pt wire were used as reference electrode and counter electrode respectively. KOH (1 M) solution was filled in a Teflon cell as electrolyte. Before the electrochemical measurement, argon was purged through the cell for 30 min to remove oxygen. The temperature of the electrolyte in the cell was kept at 25 ^{o}C using a water circulation system. Working electrodes were fabricated by depositing target materials on glass carbon (GC) electrodes (PINE, 5 mm diameter, 0.196 cm^2 area). Before depositing materials, a thorough surface clean was performed by polishing the GC electrodes with Al_2O_3 suspension (5 and 0.25 μm, Allied High Tech Products, Inc.). Afterwards, 4.8 mg of powder sample was dispersed in a mixed solution of 0.75 mL of H_2O, 0.25 mL of 2-propanol and 50 μL of Nafion (5% in a mixture of water and alcohol). Then the mixture solution was treated in sonication bath for 30 min to form a homogeneous ink. After that, 5.25 μL of catalyst ink was dropped onto the GC electrode and dried under light irradiation for 10 min. The catalyst loading was calculated to be 0.12 mg/cm^2 in all cases for GC electrodes.

The linear sweep voltammetry (LSV) curves were collected by sweeping the potential from 0.7 V to 1.7 V vs RHE with a scan rate of 10 mV/s. Cyclic voltammetry (CV) measurements were carried out in a potential range between 0.7 and 1.6 vs RHE with a scan rate of 50 mV/s. In all measurements, the IR drop was compensated at 85%, and a rotating disc electrode configuration was kept a rotation speed of 2000 rpm. To perform stability tests, an electrode was firstly fabricated by drop-casting the catalyst ink on the surface of pre-washed nickel foam (0.5×1 cm^2) with 3.5 M HCl solution in an ultrasound bath for 10 min. A catalyst loading was around 1 mg/cm^2. Then, the potential was recorded on this catalyst@Ni foam electrode at a constant current of 5 mA over a period of 18 h electrolysis.

Characterization

Powder X-ray diffraction (XRD) patterns were collected at room temperature on a Stoe theta/theta diffractometer in Bragg-Brentano geometry (Cu $K\alpha_{1/2}$ radiation) with a secondary monochroamtor. Data were measured with a proportional counter working as a point detector. Transmission electron microscopy (TEM) images of samples were measured at 100 kV by an H-7100 electron microscope from Hitachi. High resolution TEM (HR-TEM) and scanning electron microscopy (SEM) images were taken on HF-2000 and Hitachi S-5500 microscopes, respectively. N_2-sorption isotherms were measured using 3Flex Micrometrics at 77 K. Prior to the measurements, the samples were degassed at 150 °C for 10 h. Brunauer-Emmett-Teller (BET) surface areas were determined from the relative pressure range between 0.06 and 0.2. X-ray photoelectron spectroscopy (XPS) measurements were carried out with a SPECS GmbH spectrometer with a hemispherical analyzer (PHOIBOS 150 1D-DLD). The monochromatized Al Kα X-ray source (E = 1486.6 eV) was operated at 100 W. An analyzer pass energy of 20 eV was

applied for the narrow scans. The medium area mode was used as lens mode. The base pressure during the experiment in the analysis chamber was 5 x 10^{-10} mbar. The binding energy scale was corrected for surface charging by use of the C 1s peak of contaminant carbon as reference at 284.5 eV. The Fourier transform infrared spectroscopy was collected on PerkinElmer UATR Two using a diamond crystal, the number of scan was 32 and the resolution was 4 cm^{-1}. Ultraviolet-visible (UV-vis) extinction spectroscopy was used to determine the light extinction properties of the initial cobalt oxide powders dispersed in water. Extinction measurements were performed in transmission by using an extinction-calibrated spectrometer (Evolution 201, Thermo Fisher Scientific) and a quartz glass cuvette with 10 mm beam path. All samples were prepared equally for the measurement. The mass concentration of the powders in water was identical (0.0033 vol.-%). Before each measurement, the metastable dispersions were treated with ultrasonic for 1 min.

Synchrotron diffraction data were collected at the high resolution powder diffraction beamline (P02.1) at PETRA III (DESY). Samples were filled into 0.5 mm glass capillaries and mounted on the instrument sample spinner. Data were collected at 60 keV (λ=0.207200 Å) with a Perkin Elmer XRD1621 area detector. Rietveld refinements were performed with the program package TOPAS V6, BRUKER AXS.

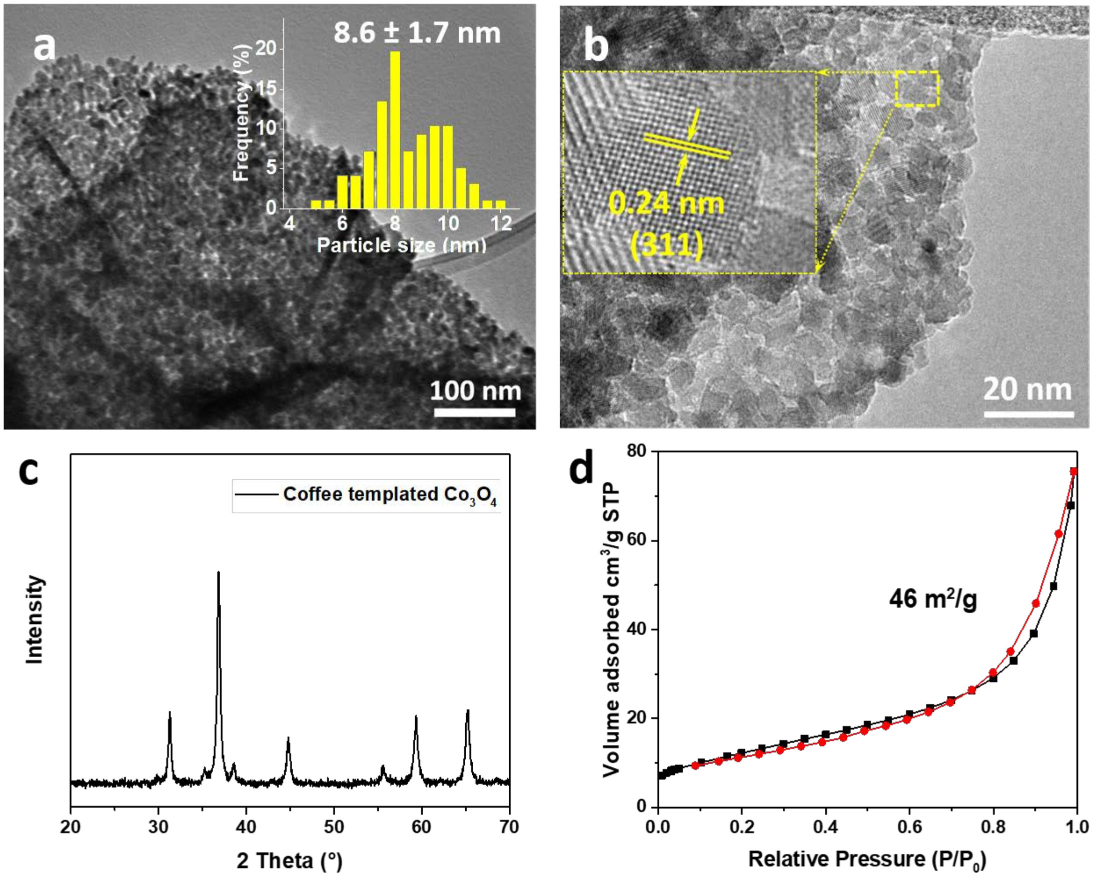

Figure S1. (a) TEM images and corresponding particle size distribution histogram, (b) high-resolution TEM image, (c) XRD patterns, and (d) N_2-sorption isotherms (d) of coffee waste templated Co_3O_4. From coffee waste templating, spinel Co_3O_4 was obtained with particles size of ~8.6 nm and BET surface area of 46 m^2/g. Inset in (b) is the corresponding close-up of the marked rectangle (yellow color), where clear crystal planes with a spacing of 0.24 nm is displayed, corresponding to the (311) lattices of the Co_3O_4 spinel (PDF card: 42-1467).

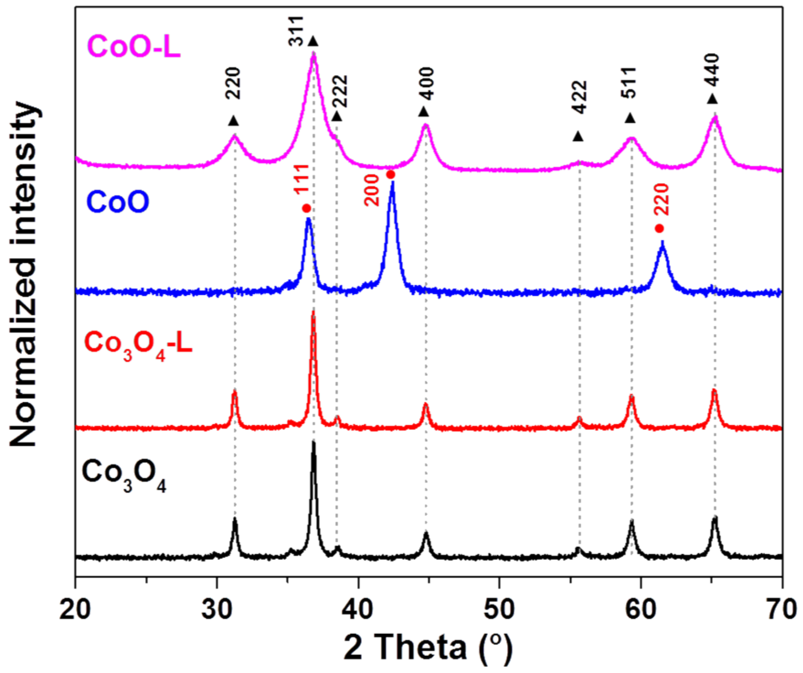

Figure S2. Wide-angle XRD patterns of Co_3O_4, CoO and their products after pulsed laser fragmentation. Black triangle: Co_3O_4 (PDF: 42-1467), red dot: CoO (PDF: 43-1004).

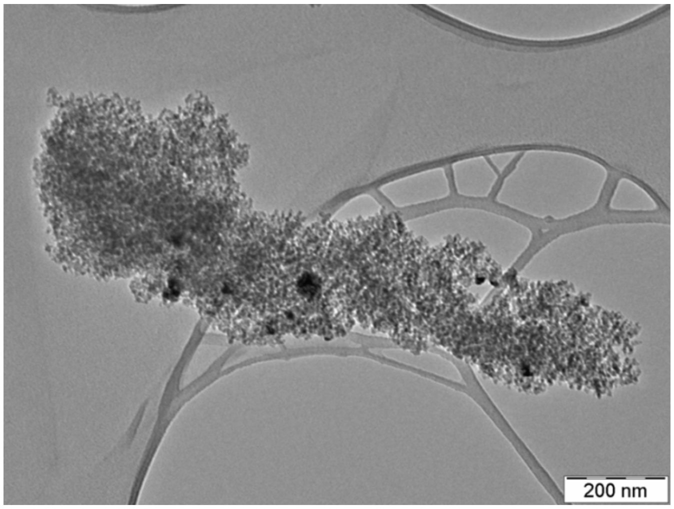

Figure S3. TEM image of Co_3O_4-L. No obvious change can be observed in the morphology after performing the PLFL process on Co_3O_4.

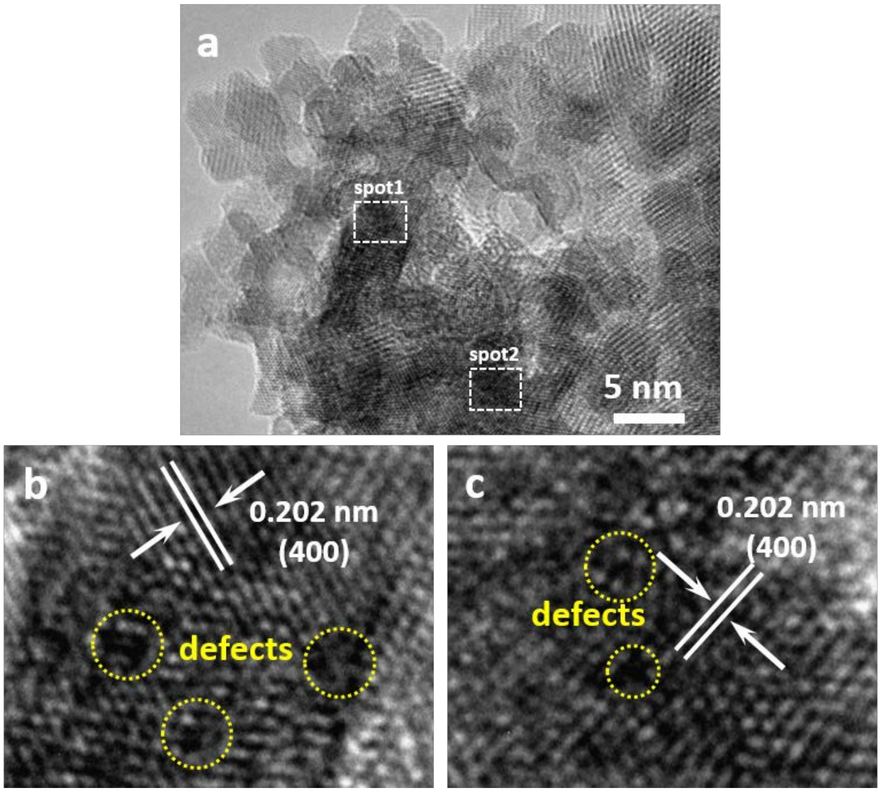

Figure S4. (a) HR-TEM of CoO-L. (b) and (c) are the corresponding close up of spot 1 and spot 2 in (a). The yellow circles represent structural defects in CoO-L from laser irradiation. Clear crystal planes with a spacing of 0.202 nm is displayed in Figure S4a-b, which can be indexed to the (400) lattices of the Co_3O_4 spinel (PDF card: 42-1467).

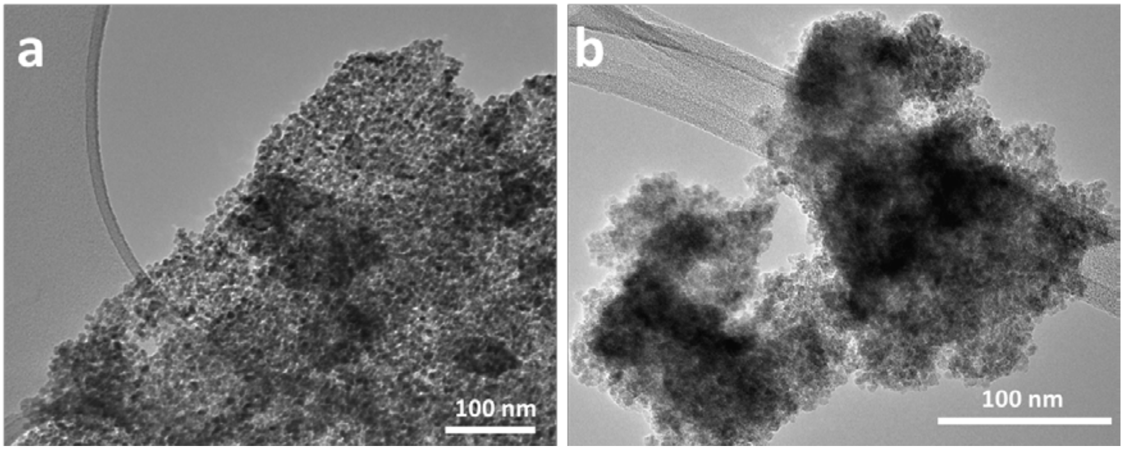

Figure S5. TEM images of (a) CoO and CoO-L (b) for determining the average particle size with 120 counts.

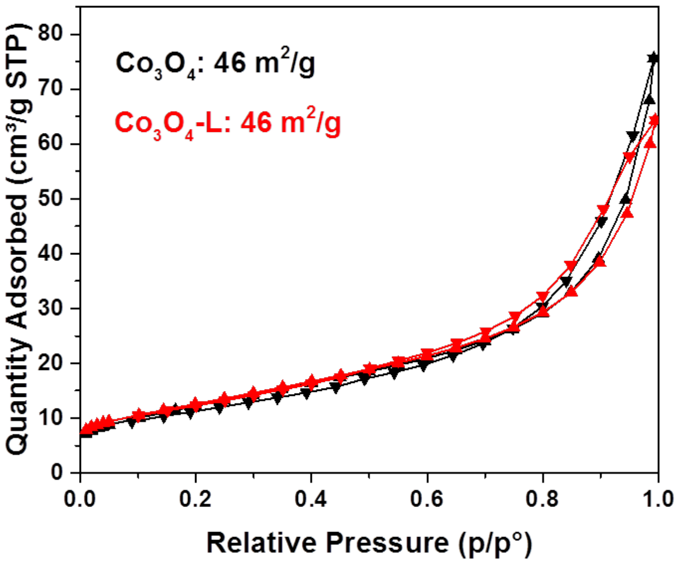

Figure S6. Nitrogen sorption isotherms of Co_3O_4 and Co_3O_4-L.

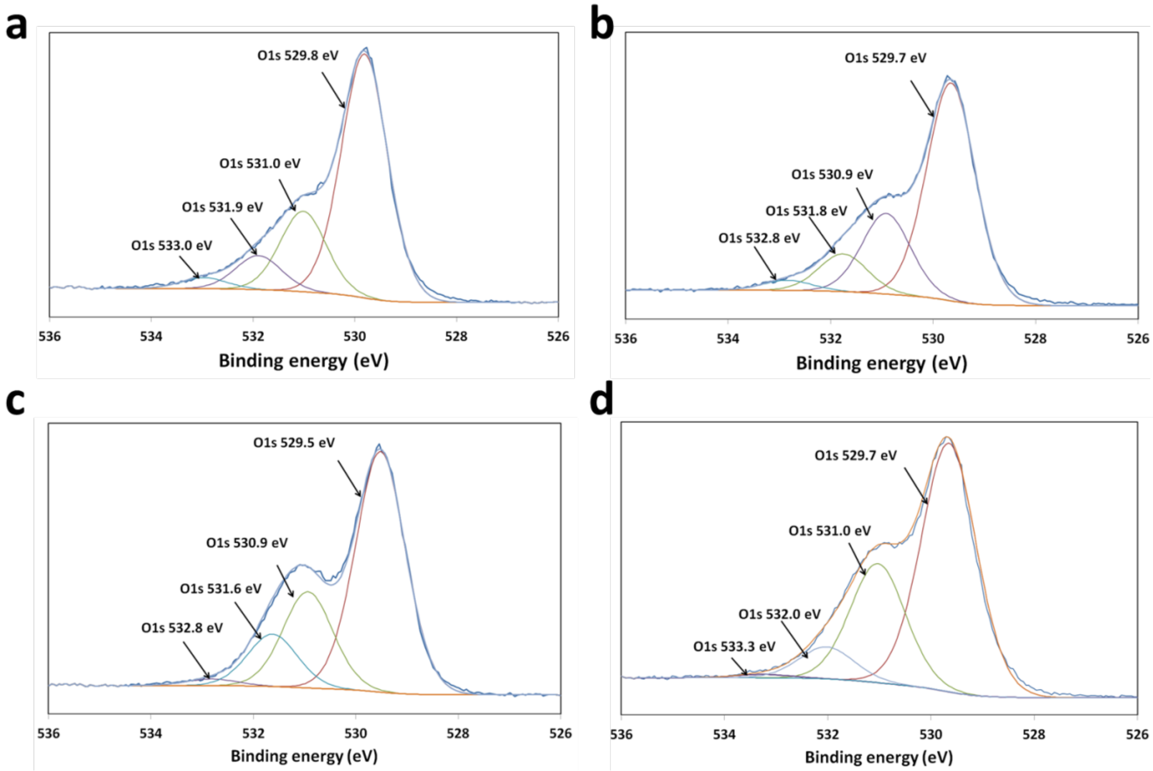

Figure S7. High resolution XPS scans of O 1s of (a) Co_3O_4, (b) Co_3O_4-L, (c) CoO, and (d) CoO-L, respectively.

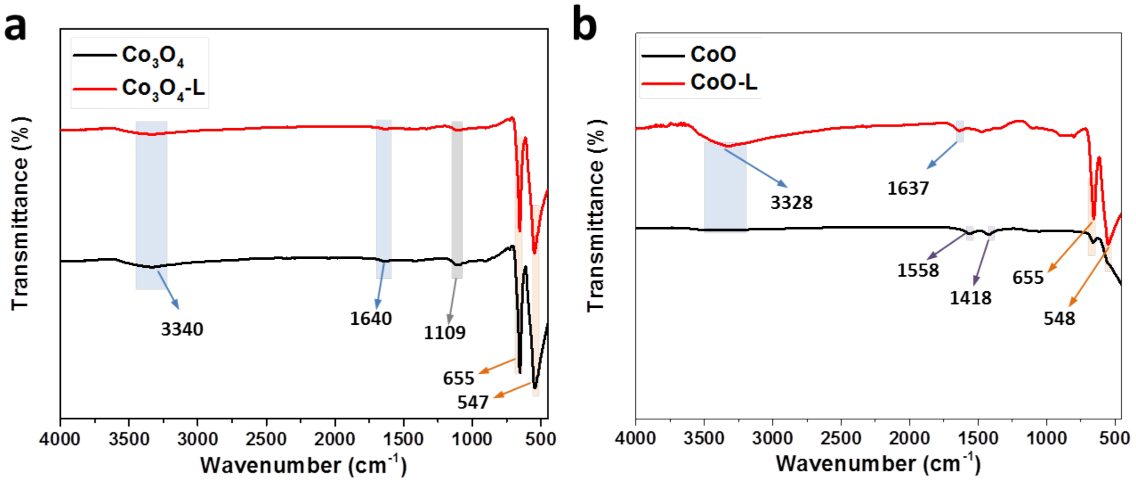

Figure S8. FT-IR spectra (a) of coffee waste templated Co_3O_4 and the product after laser fragmentation, (b) of CoO and the product collected from laser fragmentation.

As marked in the IR spectra, the sharp absorption bands at around 547 cm^{-1} and 655 cm^{-1} are associated with the stretching vibration modes of tetrahedrally and octahedrally coordinated Co-O bond, respectively. In Figure S6a, the peak at 1109 cm^{-1} belongs to vibrations of C-O. Two peaks at 1558 and 1418 cm^{-1} in CoO could be assigned to the ethyl group from ethanol reduction (Figure S6b). The absorption peak at around 1640 cm^{-1} and a broad peak at 3340 cm^{-1} are attributed the stretching vibration mode of OH^- group.[S1]

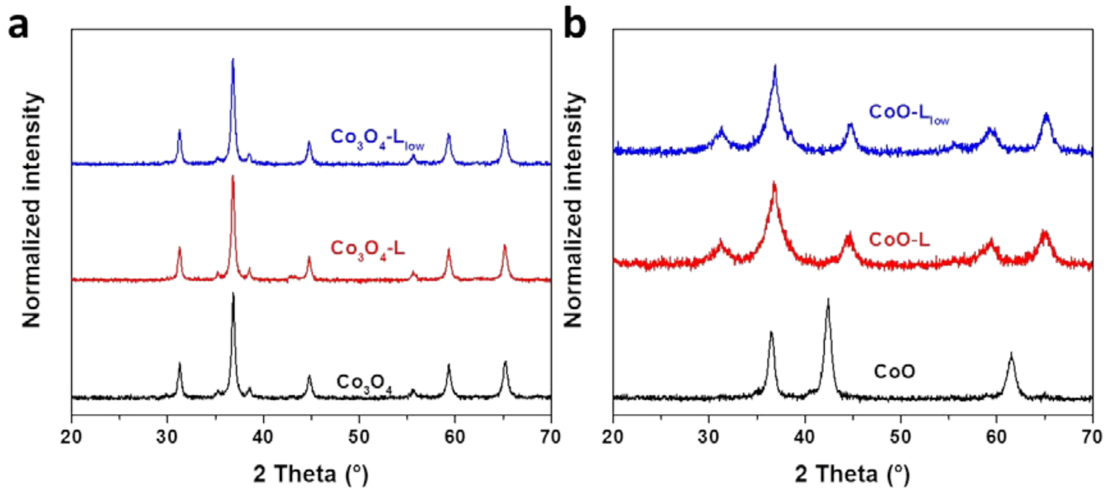

Figure S9. XRD patterns of (a) Co_3O_4 and (b) CoO, and their product after irradiated by different intensity of laser, labeled as –L and –L_{low} corresponding to laser fluence of 700 and 7 mJ/cm^{-2}, respectively.

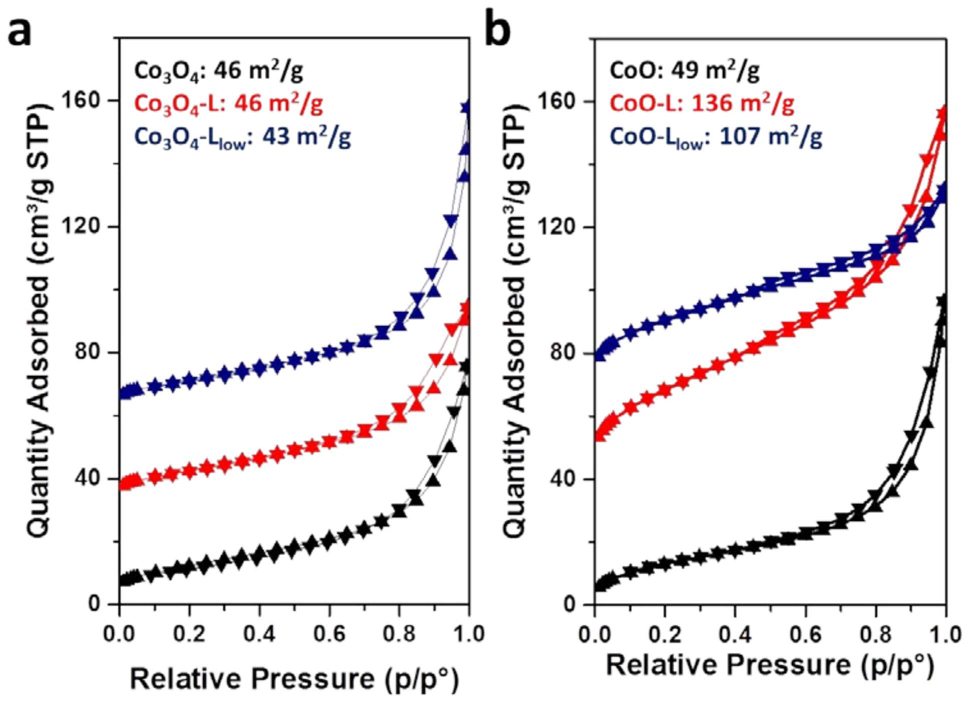

Figure S10. Nitrogen sorption isotherms of (a) Co_3O_4 and (b) CoO, and their product after irradiated by different intensity of laser.

As a comparison experiment, we conducted PLFL on cobalt oxides prepared through direct calcination of cobalt nitrate precursors, which were labelled as C-Co_3O_4 and C-CoO for the reduced one (Figure S11). Similar effects on structure could be observed on direct calcined oxides, in comparison to hard-templated cobalt oxides. A small amount of cobalt monoxide was formed on C-Co_3O_4 (Figure S12a), which was identified as smaller nanoparticles and contributed a higher surface area (Figure S12c and S13a). In the case of C-CoO, partial oxidation occcured after laser irradiation (Figure S12b). According to the refinement result of XRD patterns, 17.2 % of C-CoO was oxidized to Co_3O_4 spinel (Figure S14), instead of a much higher oxidation on CoO from hard templating. This can be explained by a shorter distance of oxygen diffusion inside R-Co_3O_4 nanoparticles (~ 8 nm) during oxidation, compared to C-CoO nanoparticles (20-50 nm). Nevertheless, much smaller particles with 3 times increase of surface area were obtained in the oxidized sample, further supporting that particle fragmentation in water goes along with oxidation process. When employed as OER electrocatalysts, increased activity was obtained on C-CoO after the PLFL process, which is still lower than that of CoO-L (Figure S15). Considering oxygen vacancies could also be generated on C-CoO, the superior OER activity of CoO-L results from larger specific surface area and higher ratio of Co^{3+}, which is regarded as the active site for cobalt oxides. This result demonstrates the advantage of preparing

cobalt oxides from coffee waste templating over solid-solid reaction for the following PLFL process.

To exclude the effect of the laser absorption properties on each sample, ultraviolet-visible spectroscopy was conducted on suspensions of the different cobalt oxide powders (Figure S16). Although Co_3O_4 colloids extinct more light at the laser wavelength of 532 nm compared to CoO, the laser absorption is more related to the particle size, since large Co_3O_4 particles showed even higher laser absorption compared to the small CoO particles. Nonetheless, significant fragmentation was observed in CoO particles despite the lower laser absorption. In summary, it is reasonable to conclude that the oxidation effect is the key factor supporting effective particle fragmentation regardless of particle size. Thermal effects seemed to play only a secondary role by initiating or accelerating the oxidation processes, since comparably low fluence and corresponding low temperatures were required to induce the fragmentation of CoO particles.

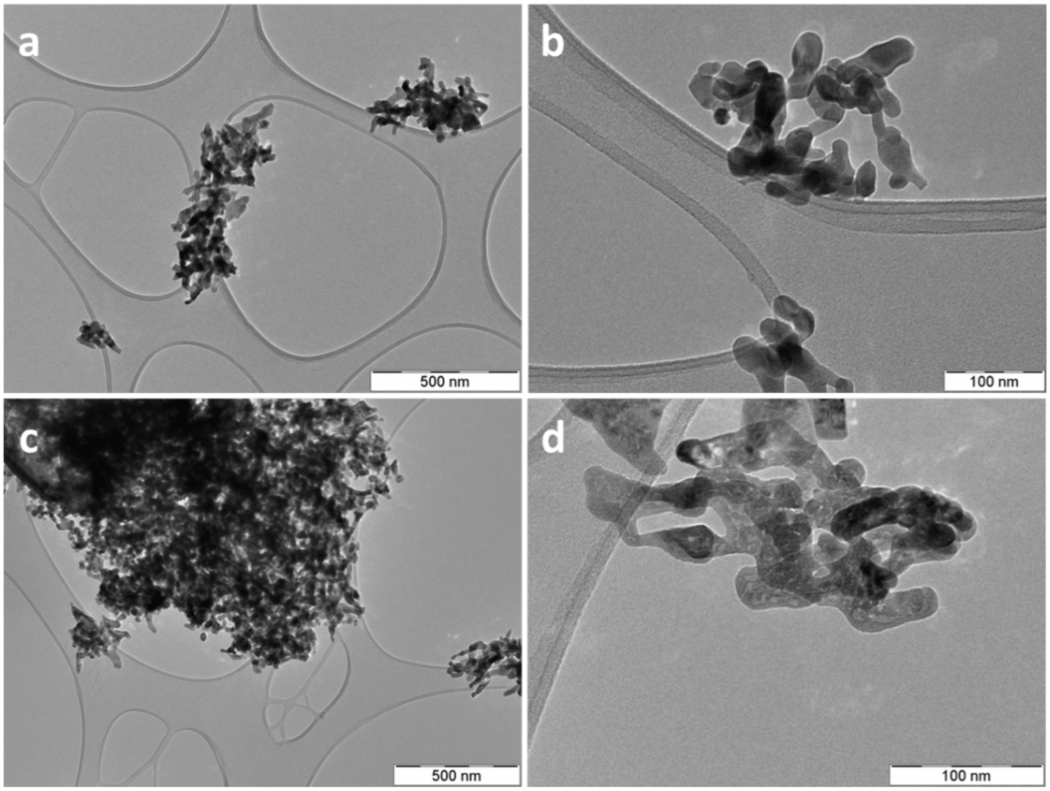

Figure S11. TEM images of (a, b) C-Co_3O_4 and (c, d) C-CoO.

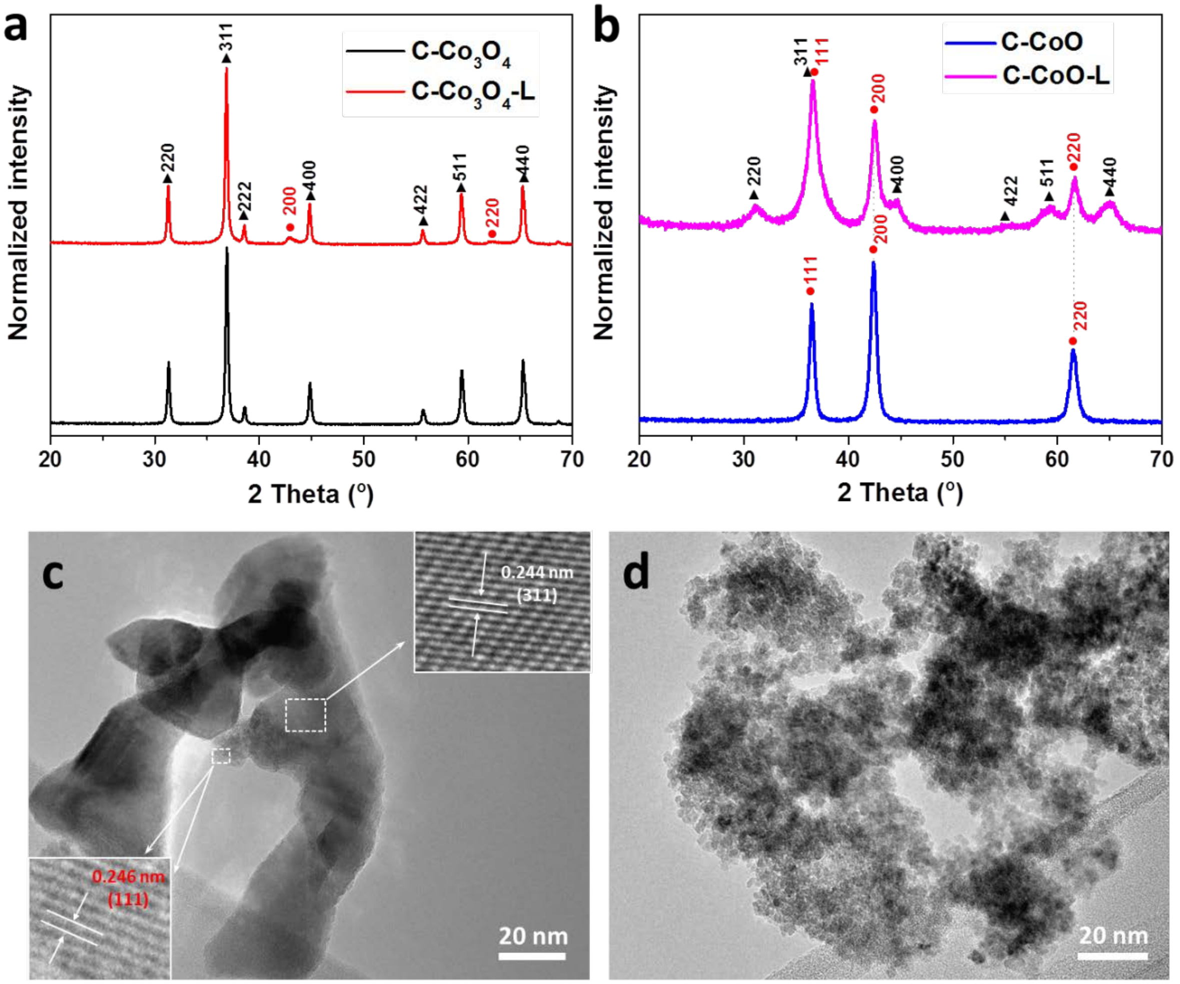

Figure S12. (a, b) Wide-angle XRD patterns of cobalt oxides from direct calcination and their products after PLFL process. Black triangle: Co_3O_4 (PDF: 42-1467), red dot: CoO (PDF: 43-1004). (c, d) TEM images of C-Co_3O_4-L and C-CoO_4-L. The inset in (c) shows the lattice planes of Co_3O_4 (white) and CoO (red).

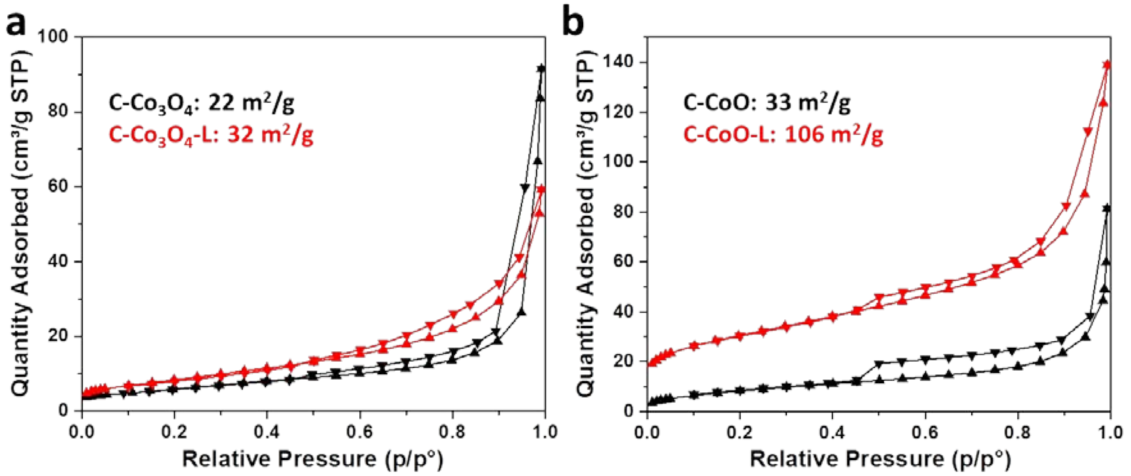

Figure S13. Nitrogen sorption isotherms of (a) C-Co_3O_4, C-Co_3O_4-L and (c, d) C-CoO, C-CoO-L.

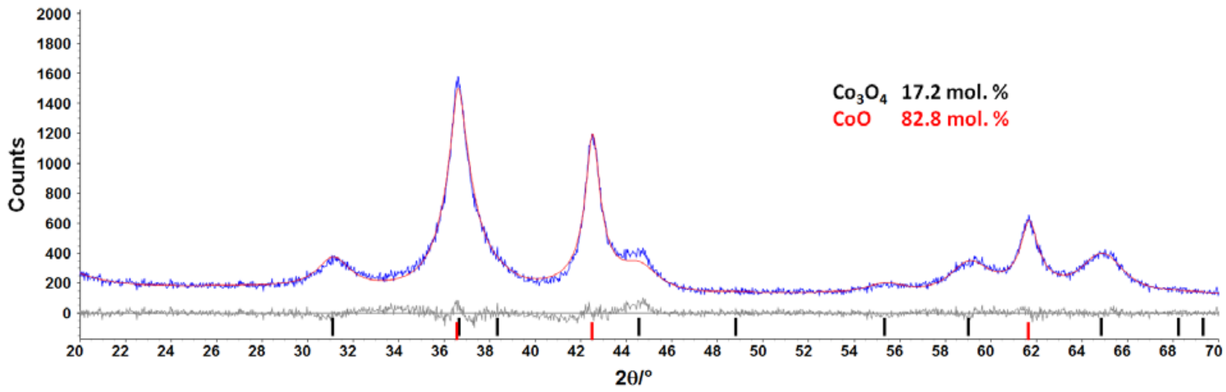

Figure S14. Rietveld refinement plot of XRD data of C-CoO-L. The mixture was calculated to be comprised of CoO (17.2 mol. %) and Co_3O_4 (82.8 mol. %).

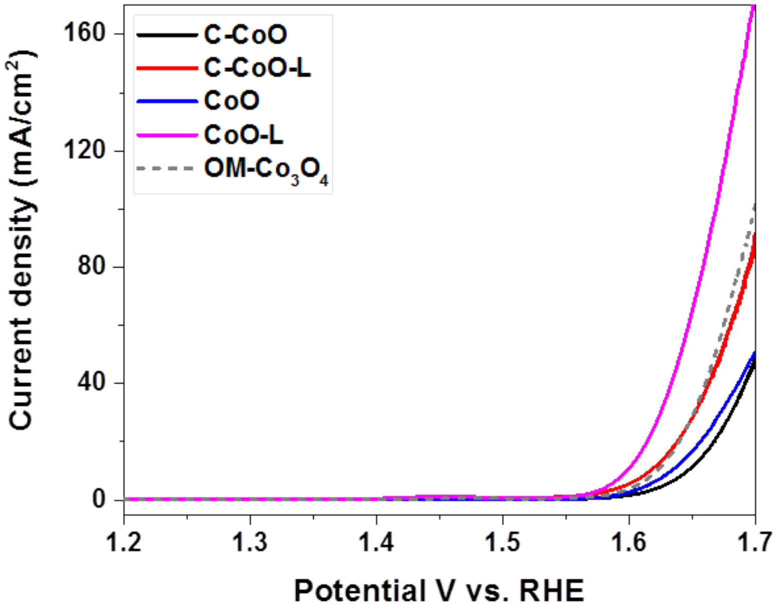

Figure S15. LSV curves of direct-calcined cobalt oxides (C-CoO) in comparison with those of coffee-waste templated CoO and nanocasted Co_3O_4 (OM-Co_3O_4).

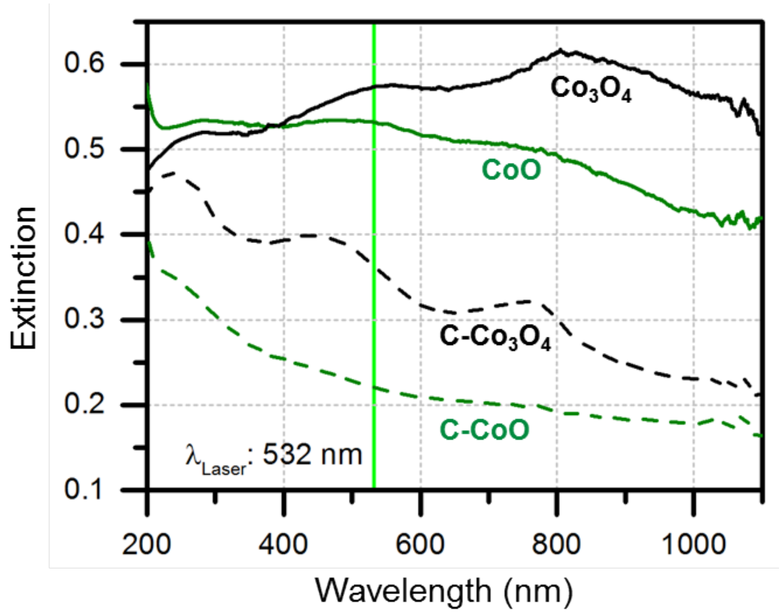

Figure S16. Ultraviolet-visible spectra of suspensions of Co_3O_4 and CoO particles through coffee-waste templating and direct calcination.

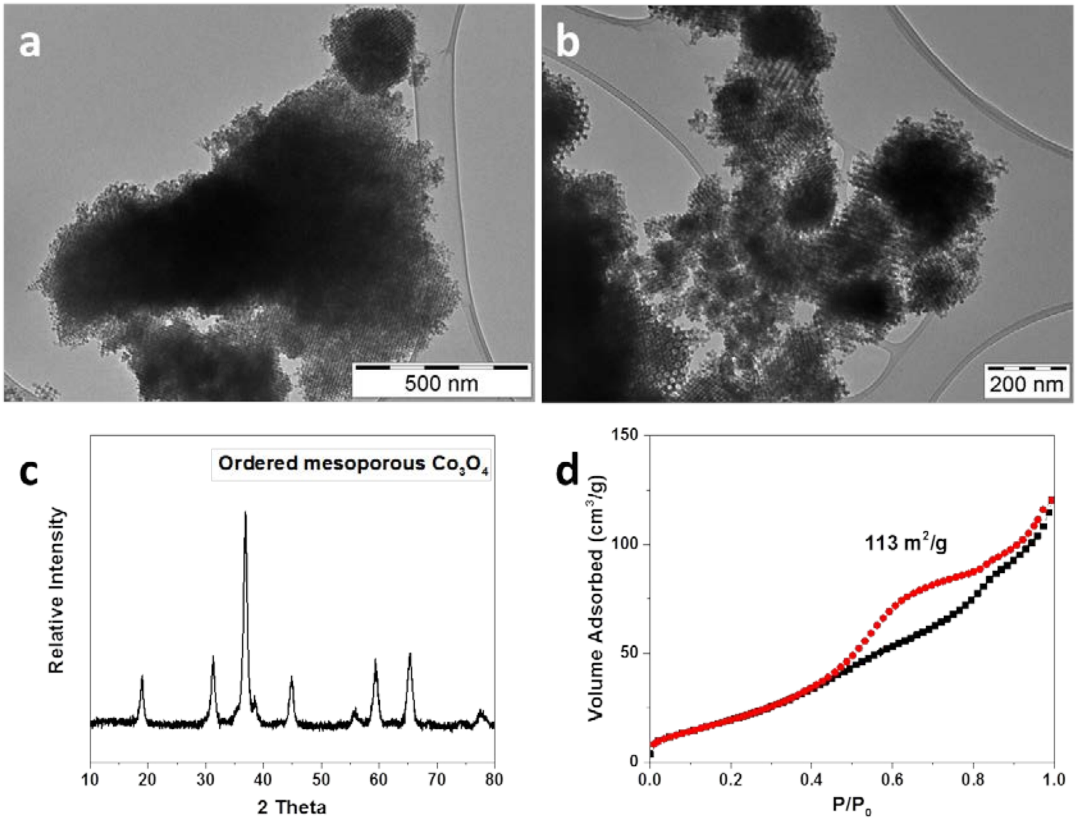

Figure S17. Structure characterization of ordered mesoporous Co_3O_4 through nanocasting: TEM images (a, b), XRD pattern (c), and nitrogen sorption isotherms (d) of OM-Co_3O_4.

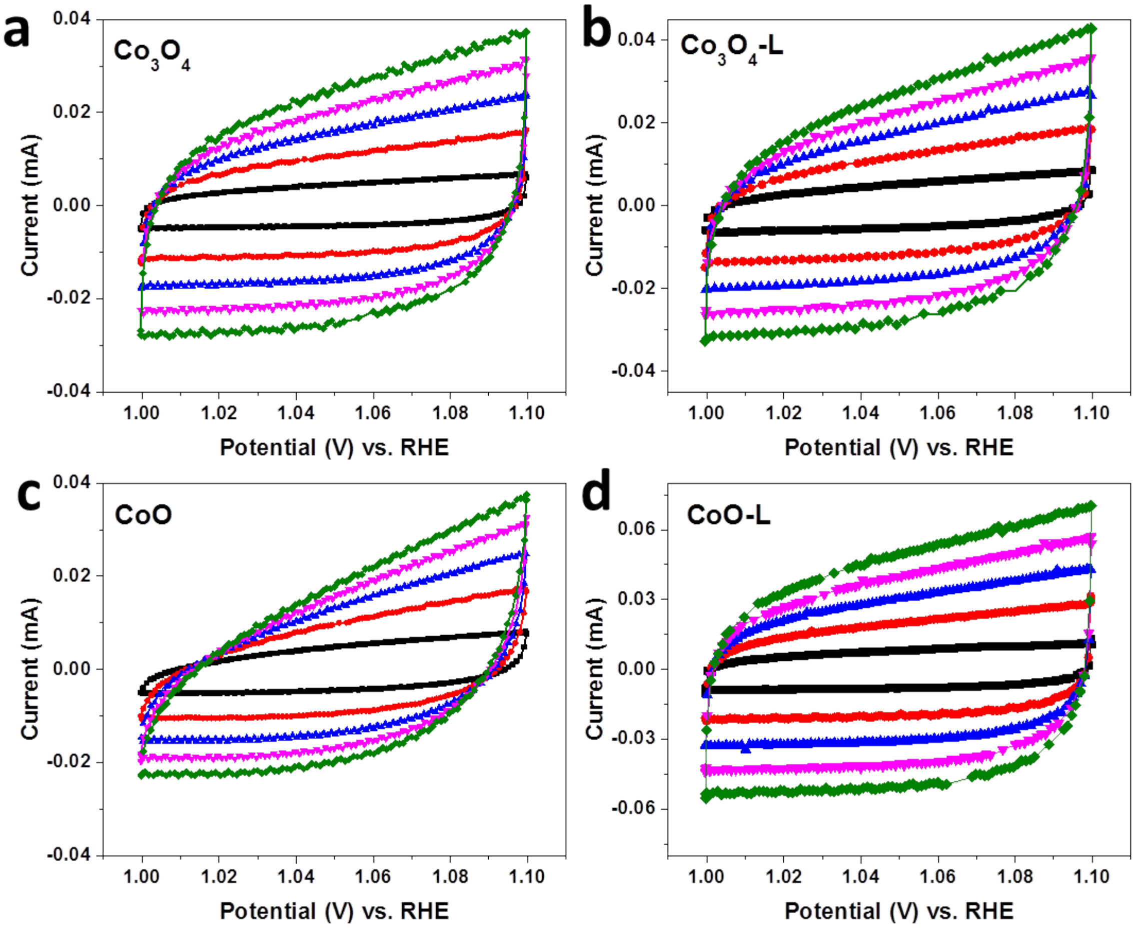

Figure S18. CV curves of (a) Co_3O_4, (b) Co_3O_4-L, (c) CoO, and (d) CoO-L, measured in a non-Faradaic region (1 - 1.1 V vs RHE) at the following scan rates: 20 mV/s (black), 60 mV/s (red), 100 mV/s (blue), 140 mV/s (magenta), and 180 mV/s (olive).

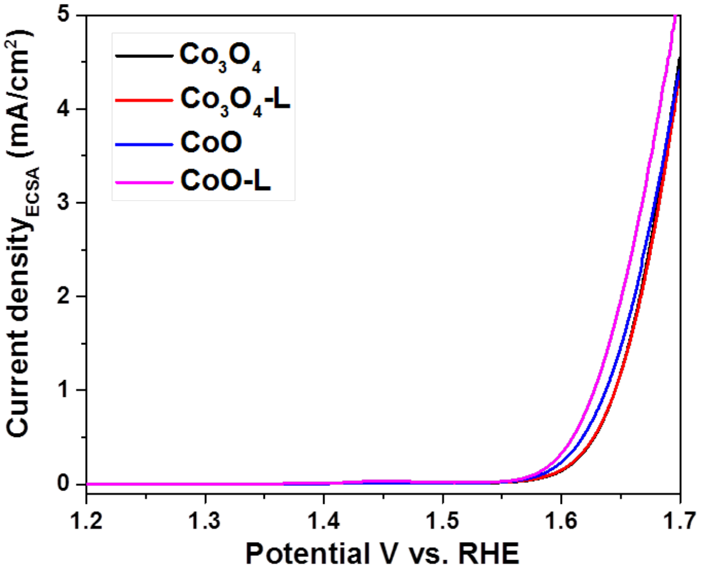

Figure S19. LSV curves normalized to the ECSA for the cobalt oxide electrocatalysts.

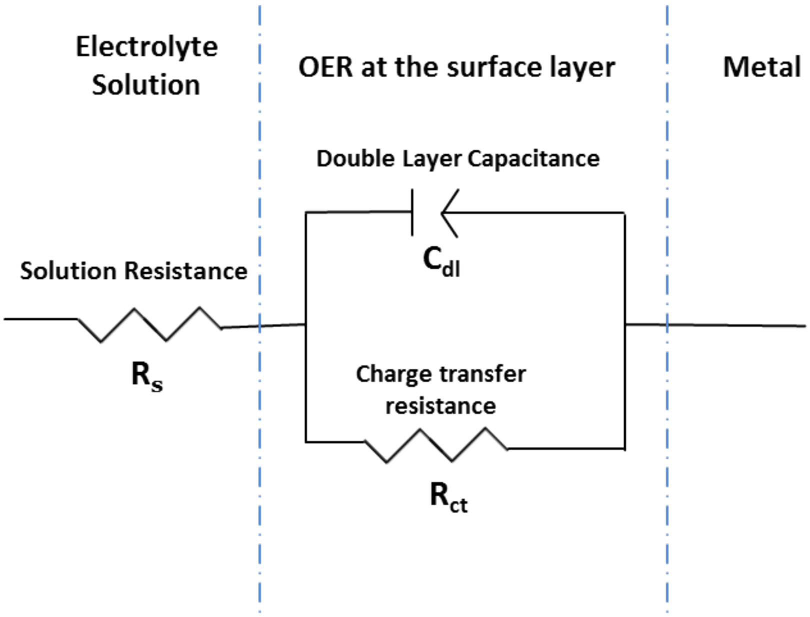

Figure S20. Simplified Randles circuit for the metal oxides catalyzing OER.

In a simplified Randles circuit, R_s represents the solution resistance, while C_{dl} element models the double-layer capacitance. The kinetics for Faradaic OER is determined by charge transfer resistance (R_{ct}).

Table S1. Results obtained from the Rietveld analyses of HR-XRD data.

	Co_3O_4	Co_3O_4-L		CoO		CoO-L	
**Unit cell parameter (Å)**	8.0919(1)	8.0961(1)		8. 4.2589(1)		8.0987 (4)	
**Composition**	Co_3O_4	8 wt. % CoO	92 wt. % Co_3O_4	95 wt. % CoO	5 wt. % Co_3O_4	5 wt. % CoO	95 wt. % $Co_{0.9}Co_2O_{4-x}$

Table S2. Summary of binding energies (B.E.) based on the curve-fitted XPS in Co 2p region.

Sample	Co $2p_{3/2}$				Co $2p_{1/2}$	
	Co^{3+}	Co^{2+}	Co^{2+} sat.	Co^{2+} sat.	Co^{3+}	Co^{2+}
**Co_3O_4**	779.7	781.3	785.6	789.5	794.9	796.5
**Co_3O_4-L**	779.7	781.4	785.9	789.5	794.9	796.6
**CoO**	-*	780.1	786.1	-	-	795.8
**CoO-L**	779.7	781.4	785.4	789.6	794.9	796.6

*: XPS is a surface technique; small amount of Co_3O_4 could remain in the particle core which could not be detected by XPS.

Table S3. Binding energies (B.E.) and Area percentages (A.E.) of different oxygen species based on the curve-fitted XPS binding energies in O 1s region.

Sample	lattice O		lattice -OH		adsorbed -OH		surface -O-C=O	
	B.E. (eV)	A.E. (%)	B.E. (eV)	A.E. (%)	B.E. (eV)	A.E. (%)	B.E. (eV)	A.E. (%)
**Co_3O_4**	529.8	66.1	531.0	21.7	531.9	9.2	533.0	3.0
**Co_3O_4-L**	530.0	63.0	530.9	23.3	531.8	10.8	532.8	2.9
**CoO**	530.0	60.8	530.9	24.3	531.6	13.2	532.8	1.7
**CoO-L**	529.7	61.7	531.0	29.5	532.0	7.9	533.3	0.9

Table S4. Summarized electrochemical data for cobalt oxide elextrocatalysts.

	Co_3O_4	Co_3O_4-L	CoO	CoO-L	OM-Co_3O_4
η@10 mA/cm^2 (mV)	402	400	403	369	392
j@1.7 V vs. RHE (mA/cm^2)	75.2	78.1	50.6	172.2	102.0
Tafel slope (mV/dec)	51	52	51	46	53
C_{dl} (mF)	0.13	0.14	0.09	0.26	-
ECSA (cm^2)	3.25	3.5	2.25	6.5	-
R_s (ohm)	6.2	6.1	6.6	5.7	-
R_{ct} (ohm)	48.8	38.3	25.8	12	-

Table S5. OER performanc of cobalt based electrocatalysts reported in recent literatures.

Catalyst	Electrolyte	Loading (mg/cm^2)	Tafel slope (mV/dec)	η at *j* = 10 mA/cm^2 (mV)	Reference
Co_3O_4/N-rmGO@Ni foam	0.1 M KOH	1	42	310	S2
$Co(NO_3)_2$ $6H_2O$	1 M KOH	0.12	55.9	323	S3
$CoFe_2O_4$/CoO	1 M KOH	0.2	71	320	S4
Co_3O_4/$NiCo_2O_4$@Ni foam	1 M KOH	1	88	340	S5
Co_3O_4 NPs/graphene	1 M KOH	0.19	56	350	S6
$Co_{2.25}Cr_{0.75}O_4$	1 M NaOH	0.84	60 ± 3	350 ± 10	S7
CoO-L	1 M KOH	0.12	46	369	This work
$CoFe_2O_4$ nanorods	1 M KOH	0.19	96	355	S8
STL templated Co_3O_4	1 M KOH	0.12	45-53	401	S9
Reduced Co_3O_4 nanowires	1 M KOH	0.136	72	400	S10
Nanoparticulate Co_3O_4	1 M NaOH	0.8	60.9	500 ± 10	S11

Reference:

S1. N. Shi, W. Cheng, H. Zhou, T. Fan, M. Niederberger, *Chem. Commun*. **2015**, *51*, 1338-1340.

S2. Y. Liang, Y. Li, H. Wang, J. Zhou, J. Wang, T. Regier, H. Dai, *Nat. Mater*. **2011**, *10*, 780-786.

S3. G. Moon, M. Yu, C. K. Chan, H. Tüysüz, *Angew. Chem*. **2019**, *58*, 3491-3495

S4. F. Waag, B. Gökce, C. Kalapu, G. Bendt, S. Salamon, J. Landers, U. Hagemann, M. Heidelmann, S. Schulz, H. Wende, N. Hartmann, M. Behrens, S. Barcikowski, *Sci. Rep*. **2017**, *7*, 13161.

S5. H. Hu, B. Guan, B. Xia and X. W. Lou, *J. Am. Chem. Soc*. 2015, 137, 5590-5595.

S6. Y. Zhao, S. Chen, B. Sun, D. Su, X. Huang, H. Liu, Y. Yan, K. Sun, G. Wang, *Sci. Rep*. **2015**, *5*, 7629.

S7. C.-C. Lin, C. C. L. McCrory, *ACS Catal*. **2017**, *7*, 443-451.

S8. Y. Ding, J. Zhao, W. Zhang, J. Zhang, X. Chen, F. Yang, X. Zhang, *ACS Appl. Energy Mater*. **2019**, *2*, 1026-1032.

S9. X. Deng, C. K. Chan, H. Tüysüz, *ACS Appl. Mater. Interfaces* **2016**, *8*, 32488–32495.

S10. Y. Wang, T. Zhou, K. Jiang, P. Da, Z. Peng, J. Tang, B. Kong, W.-B. Cai, Z. Yang, G. Zheng, *Adv. Energy Mater*. **2014**, *4*, 1400696.

S11. F. Song, X. Hu, *J. Am. Chem. Soc*. **2014**, *136*, 16481-16484.
